# Role of heme oxygenase-1 in the pathogenesis and tumorigenicity of Kaposi's sarcoma-associated herpesvirus

**DOI:** 10.18632/oncotarget.7227

**Published:** 2016-02-07

**Authors:** Lu Dai, Jing Qiao, David Nguyen, Amanda P. Struckhoff, Lisa Doyle, Karlie Bonstaff, Luis Del Valle, Chris Parsons, Bryan P. Toole, Rolf Renne, Zhiqiang Qin

**Affiliations:** ^1^ Research Center for Translational Medicine and Key Laboratory of Arrhythmias, East Hospital, Tongji University School of Medicine, Shanghai, China; ^2^ Departments of Microbiology/Immunology/Parasitology, Louisiana State University Health Sciences Center, Louisiana Cancer Research Center, New Orleans, LA, USA; ^3^ Department of Medicine, Louisiana State University Health Sciences Center, Louisiana Cancer Research Center, New Orleans, LA, USA; ^4^ Department of Pediatrics, East Hospital, Tongji University School of Medicine, Shanghai, China; ^5^ William Carey University College of Osteopathic Medicine, Hattiesburg, MS, USA; ^6^ Department of Pathology, Louisiana State University Health Sciences Center, Louisiana Cancer Research Center, New Orleans, LA, USA; ^7^ Department of Regenerative Medicine & Cell Biology, Medical University of South Carolina and Hollings Cancer Center, Charleston, SC, USA; ^8^ Department of Molecular Genetics Microbiology, University of Florida, Gainesville, FL, USA

**Keywords:** KSHV, Kaposi's sarcoma, HO-1, SnPP

## Abstract

Kaposi's Sarcoma-associated Herpesvirus (KSHV) is the etiologic agent of several malignancies, including Kaposi's Sarcoma (KS), which preferentially arise in immunocompromised patients such as HIV+ subpopulation and lack effective therapeutic options. Heme oxygenase-1 (HO-1) has been reported as an important regulator of endothelial cell cycle control, proliferation and angiogenesis. HO-1 has also been found to be highly expressed in KSHV-infected endothelial cells and oral AIDS-KS lesions. We previously demonstrate that the multifunctional glycoprotein CD147 is required for KSHV/LANA-induced endothelial cell invasiveness. During the identification of CD147 controlled downstream genes by microarray analysis, we found that the expression of HO-1 is significantly elevated in both CD147-overexpressing and KSHV-infected HUVEC cells when compared to control cells. In the current study, we further identify the regulation of HO-1 expression and mediated cellular functions by both CD147 and KSHV-encoded LANA proteins. Targeting HO-1 by either RNAi or the chemical inhibitor, SnPP, effectively induces cell death of KSHV-infected endothelial cells (the major cellular components of KS) through DNA damage and necrosis process. By using a KS-like nude mouse model, we found that SnPP treatment significantly suppressed KSHV-induced tumorigenesis *in vivo*. Taken together, our data demonstrate the important role of HO-1 in the pathogenesis and tumorigenesis of KSHV-infected endothelial cells, the underlying regulatory mechanisms for HO-1 expression and targeting HO-1 may represent a promising therapeutic strategy against KSHV-related malignancies.

## INTRODUCTION

Kaposi sarcoma-associated herpesvirus (KSHV) represents a principal causative agent of cancers arising in immunocompromised patients, such as Kaposi's Sarcoma (KS) [1]. In some Acquired Immunodeficiency Syndrome (AIDS) pandemic counties of Africa, KS has become one of the commonest cancers affecting men and children with significant morbidity and mortality [2-5]. Although the incidence of AIDS-associated KS (AIDS-KS) in the Western world has declined since the widespread implementation of combined antiretroviral treatment (cART), up to 50% of patients with AIDS-KS never achieve total remission [6]. In addition, the issues of KS in the context of immune reconstitution inflammatory syndrome (IRIS) and its impact on cART rollout initiatives have become increasingly apparent recently [7-9]. Furthermore, although treatments for KS exist, none is curative, which requires better understanding the mechanisms for viral pathogenesis and tumorigenesis and developing more effective therapeutic strategies.

Heme oxygenases (HOs) are responsible for the oxidative cleavage of the heme ring, the rate-limiting step in heme catabolism [10]. Enzymatic degradation of heme releases carbon monoxide (CO), free iron, and biliverdin, which is subsequently converted to bilirubin by biliverdin reductase [10]. So far, 3 mammalian isoforms of HO have been identified: the stress-inducible HO-1 and the constitutive HO-2 and HO-3. Among them, HO-1 is strongly and rapidly up-regulated by noxious stimuli leading to oxidative stress such as transitional metals, glutathione-depleting agents and heat shock [11]. HO-1 has been recently defined as an important regulator of endothelial cell cycle control, proliferation, vascular endothelial growth factor (VEGF) secretion, and angiogenesis [12]. Interestingly, a recent study has shown the elevated HO-1 expression and activity in KSHV-infected endothelial cells as well as oral AIDS-KS lesions [13]. Another study has shown that targeting HO-1 by shRNA and chemical inhibitor, tin protoporphyrin IX (SnPP), can impair KSHV-encoded G protein-coupled receptor (vGPCR)-induced survival, proliferation, transformation and tumor growth [14]. However, the vGPCR ectopic expressed cells cannot completely represent whole virus infection situation either *in vitro* or *in vivo*. During KSHV *de novo* infection, only a small proportion of infected cells expressing vGPCR, since it is a lytic protein while most cells are in latency. Another remaining question is that the mechanisms for KSHV activation of HO-1 through either viral proteins or host factors still remain largely unknown.

The multifunctional transmembrane protein, CD147, also known as Emmprin or Basigin, induces the expression and secretion of multiple matrix metalloproteinases (MMPs), thereby promoting tumor cell invasion and other malignant behaviors [15, 16]. We recently reported that enhancement of invasiveness in primary endothelial cells (the major cellular components of KS), following *de novo* KSHV infection, results from upregulation of CD147 by the KSHV-encoded latency-associated nuclear antigen (LANA) protein [17]. Our recent microarray data indicate that as one of CD147 potentially controlled downstream candidates, the transcription of *HO-1* gene is significantly elevated in both CD147-overexpressing and KSHV-infected human umbilical vein endothelial cells (HUVEC) (25.8 and 2.31 folds, respectively) [18]. Therefore, in the current study we will continue to experimentally validate the regulation of HO-1 by CD147 and viral latent protein, investigate the role of HO-1 in KSHV-infected endothelial cell pathogenesis and tumorigenesis, and determine the anti-cancer effects of a HO-1 selective inhibitor by using an established KS-like xenograft model.

## RESULTS

### KSHV infection upregulates HO-1 expression through CD147 *in vitro* and *in vivo*

**Figure 1:**
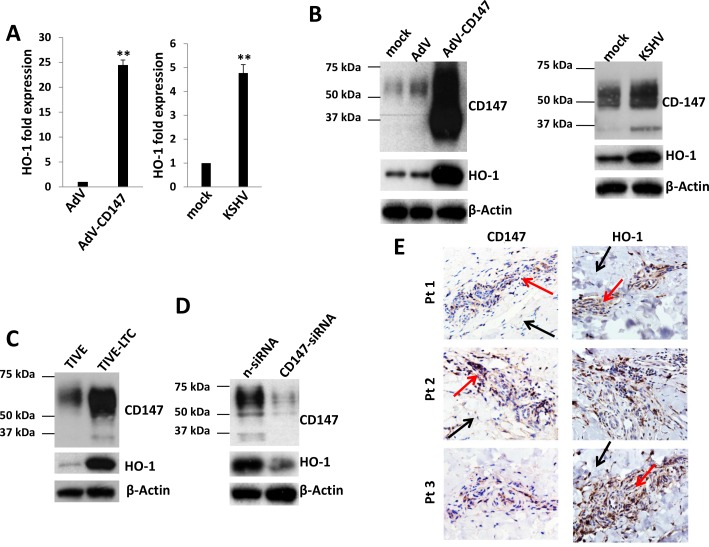
KSHV infection upregulates HO-1 expression through CD147 *in vitro* and *in vivo* **A.**-**B.** HUVEC were transduced using a recombinant human CD147-encoding adenovirus (AdV-CD147), or control adenovirus (AdV) for 48 h, or infected by purified KSHV (MOI ∼ 10) for 48 h. Gene transcription and protein expression were measured by qRT-PCR and immunoblots, respectively. Error bars represent the S.E.M. for 3 independent experiments. ** = *p* < 0.01. **C.**-**D.** Protein expression within KSHV stably infected TIVE-LTC and non-infected parental TIVE was compared by immunoblots. Some TIVE-LTC were transfected with negative control siRNA (n-siRNA) or *CD147*-siRNA for 48 h, prior to immunoblots. **E.** The expression of CD147 and HO-1 within KS tumor tissues from 3 cohort HIV-infected patients was detected by immunohistochemistry (400x magnification). Red arrows indicate the KS tumor area and black arrows indicate the adjacent normal area from the same patient.

We first used qRT-PCR to validate the microarray data as mentioned above. Our results indicated that the transcriptional level of *HO-1* was increased ∼25 and ∼4.5 folds in CD147-overexpressing and KSHV-infected HUVEC, respectively (Figure 1A). Moreover, the expression of HO-1 protein was also significantly upregulated in CD147-overexpressing and KSHV-infected HUVEC, when compared to the controls (Figure 1B). We next compared the expression of CD147 and HO-1 between KSHV long-term-infected telomerase-immortalized human umbilical vein endothelial (TIVE-LTC) and non-infected parental TIVE cells [19]. We found that the expressional levels of CD147 and HO-1 were much higher in TIVE-LTC than in TIVE cells (Figure 1C). Silencing of CD147 by RNAi greatly reduced HO-1 expression in TIVE-LTC and KSHV-infected HUVEC (Figure 1D and S1). Furthermore, we found significantly elevated expression of CD147 and HO-1 within KS tumor tissues isolated from 3 cohort HIV+ patients when compared to adjacent normal area (Figure 1E). Taken together, our data demonstrate that KSHV upregulates HO-1 expression through CD147 in endothelial cells, and the high co-expression of these 2 proteins in AIDS-KS tissues indicating their importance to tumor development.

### Inhibition of HO-1 inducing KSHV-infected endothelial cell death is independent of apoptosis

**Figure 2:**
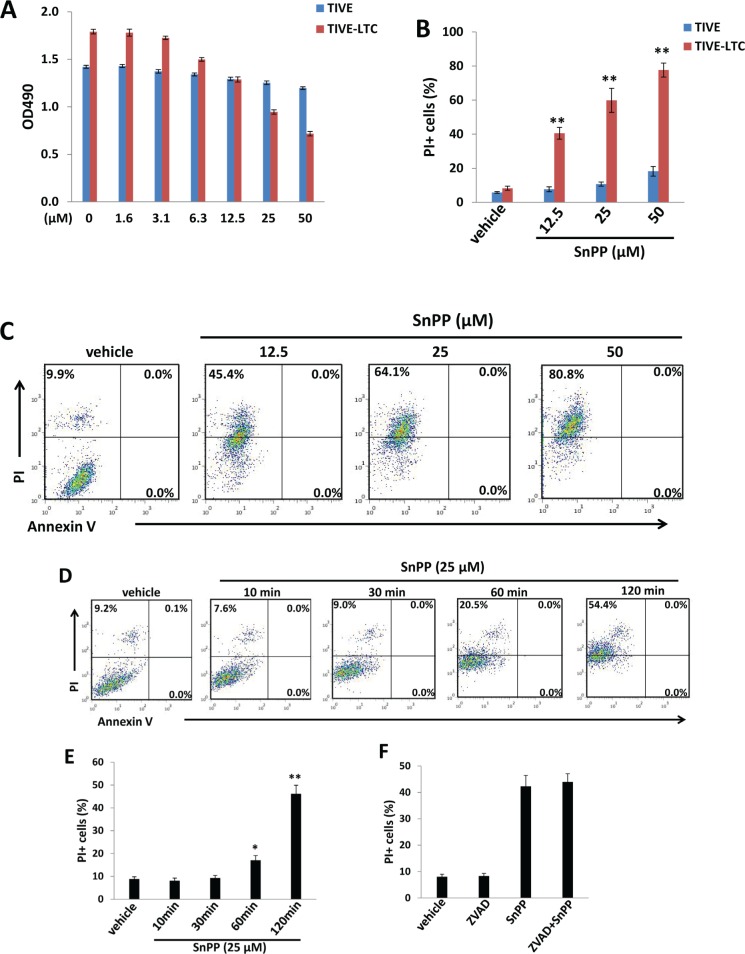
Targeting HO-1 by SnPP inducing KSHV-infected endothelial cell death is independent of apoptosis **A.** TIVE-LTC and TIVE were incubated with indicated concentrations of SnPP for 48 h, then cell proliferation was measured by the WST-1 assays as described in the Methods. **B.**-**C.** TIVE-LTC and TIVE were incubated with vehicle or indicated concentrations of SnPP for 24 h, then cell viability and apoptosis were measured by Annexin V-PI staining and flow cytometry analysis. TIVE-LTC were shown as an example for cell subpopulation diagram in panel C. **D.**-**E.** TIVE-LTC were incubated with vehicle or 25 μM of SnPP for indicated time, then cell viability and apoptosis were measured as above. **F.** TIVE-LTC were incubated with or without the pan-caspase inhibitor Z-VAD-FMK (ZVAD, 25 μM) for 2 h, followed by vehicle or 25 μM of SnPP treatment for another 2 h. Error bars represent the S.E.M. for 3 independent experiments. * = *p* < 0.05, ** = *p* < 0.01.

We next tested the effects of the HO-1 selective inhibitor, SnPP, on KSHV-infected endothelial cell growth/survival. We first confirmed the inhibition of HO-1 enzymatic activities from TIVE-LTC by SnPP treatment (Figure S2), using a biochemical assay [13, 20]. By using the WST-1 assays, we found that SnPP treatment reduced TIVE-LTC proliferation in a dose-dependent manner, whereas it only slightly reduced non-infected TIVE proliferation especially at the highest concentration (50 μM) (Figure 2A). Additionally, flow cytometry data confirmed that SnPP treatment significantly induced TIVE-LTC cell death (PI+) in a dose-dependent manner, whereas it only affected a small percentage of non-infected TIVE cells at the highest concentration (50 μM) (Figure 2B-2C). Interestingly, we did not detect any Annexin V+ cells at the time of collecting samples (Figure 2C). To determine whether this kind of cell death is really independent of apoptosis or is converted from early apoptotic cells, we collected SnPP-treated TIVE-LTC at early time points (10-120 min) and performed a similar flow cytometry analysis. Our time-course results indicated that SnPP gradually induced TIVE-LTC cell death which is indeed independent of apoptosis (no Annexin V+ cells were detected at any time-point during the period) (Figure 2D-2E). To further support the flow cytometry results, we also detected the expression of apoptosis markers including cleaved-caspase 3 and 9 [21] in these samples and found no detectable cleaved-caspases expression in SnPP-treated cells (data not shown). Also, the pan-caspase inhibitor Z-VAD-FMK [22] pretreatment cannot prevent SnPP-induced cell death in TIVE-LTC (Figure 2F). To exclude the off-target effects of SnPP causing cell death, we knocked down HO-1 by RNAi and found that it also induced significant TIVE-LTC cell death while minimally affecting TIVE cell viability (Figure S3).

### Targeting HO-1 by SnPP causes DNA damage and necrosis in KSHV-infected endothelial cells

**Figure 3:**
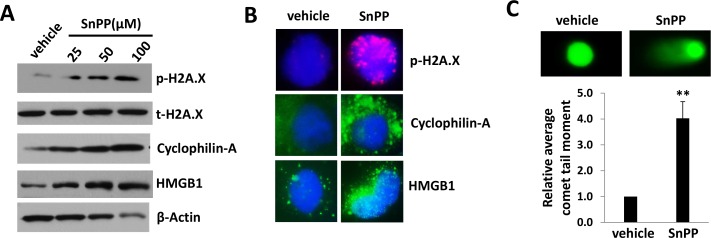
SnPP treatment causes DNA damage and necrosis for KSHV-infected endothelial cells **A.** TIVE-LTC were incubated with vehicle or indicated concentrations of SnPP for 24 h, then protein expression were measured by immunoblots. **B.**-**C.** TIVE-LTC were incubated with vehicle or 50 μM of SnPP for 24 h, then protein expression and DNA damage were measured by immunofluorescence and CometAssay, respectively. Error bars represent the S.E.M. for 2 independent experiments. ** = *p* < 0.01.

To further understand how SnPP causes cell death of TIVE-LTC, we analyzed the expression of DNA damage and necrosis markers. SnPP treatment greatly increased the expression of DNA damage marker, phosphor-H2A.X as well as two necrosis makers, Cyclophilin-A and HMGB1 [23] in TIVE-LTC as demonstrated by immunoblots analysis (Figure 3A). In comparison, we found no change of autophagy marker, LC3 [24], in SnPP-treated TIVE-LTC when compared to vehicle-treated controls (data not shown), indicating SnPP-caused cell death is not through autophagy. Immunofluorescence analysis confirmed the apparent upregulation of phosphor-H2A.X, Cyclophilin-A and HMGB1 in SnPP-treated TIVE-LTC (Figure 3B and S4). SnPP caused DNA damage was further demonstrated by CometAssay (the obvious comet tail moment in SnPP-treated TIVE-LTC when compared to vehicle-treated cells as shown in Figure 3C).

### Low doses of SnPP impair TIVE-LTC invasiveness and anchorage-independent growth

**Figure 4:**
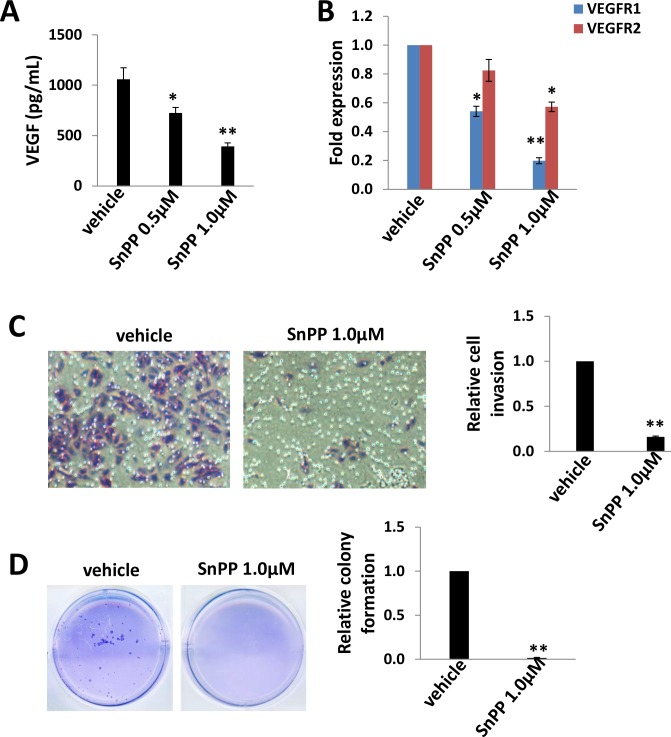
Low doses of SnPP impair TIVE-LTC invasiveness and anchorage-independent growth **A.**-**B.** TIVE-LTC were incubated with low doses of SnPP for 24 h, then the concentrations of VEGF in culture supernatants were determined using ELISA. Gene transcription was measured by qRT-PCR. **C.**-**D.** Cells were treated as above, then cell invasion and anchorage-independent growth abilities were measured by using the transwell and soft agar assays as described in the Methods. Error bars represent the S.E.M. for 3 independent experiments. * = *p* < 0.05, ** = *p* < 0.01.

Pro-angiogenic cytokines such as VEGF, are secreted by KSHV-infected cells, and their presence within KS lesions and the peripheral circulation of KS patients is thought to facilitate KSHV-associated cellular pathogenesis and angiogenesis [25, 26]. Moreover, acquisition of a migratory or invasive phenotype represents one hallmark of KSHV-infected endothelial cells, with implications for both viral dissemination and angiogenesis within KS lesions [27]. Here we found that low doses of SnPP (0.5-1.0 μM) effectively reduced VEGF production as well as VEGF receptor gene transcription in particular VEGFR1 in TIVE-LTC (Figure 4A-4B). Furthermore, we found that low doses of SnPP significantly impaired TIVE-LTC invasiveness and anchorage-independent growth, by using transwell and soft agar assays, respectively (Figure 4C-4D).

### KSHV-encoded LANA protein is responsible for upregulation of HO-1 expression

**Figure 5:**
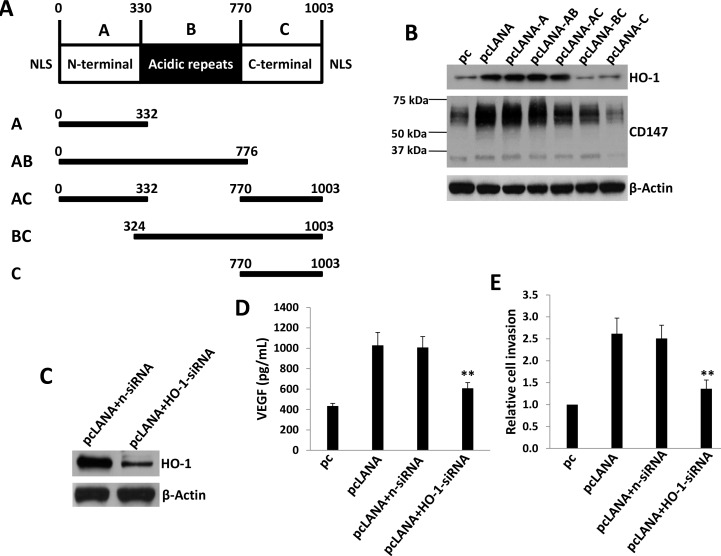
KSHV-encoded LANA protein is responsible for upregulation of HO-1 expression **A.** Putative domain structure of LANA based on primary sequence features. The N-terminal region (domain A) is rich in prolines and serines and contains a putative nuclear localization sequences (NLS). The central region of LANA (domain B) is comprised of several repeats and is very acidic. The C-terminal region (domain C) also contains a putative NLS. All fragment variants and their coordinates are depicted below the domain model of LANA. **B.** HUVEC were transfected with control vector pc, full-length LANA construct (pcLANA) and fragment variants, respectively, for 48 h. Immunoblots was used to detect protein expression. **C.**-**E.** HUVEC were first transfected with negative control siRNA (n-siRNA) or *HO-1*-siRNA for 48 h, then transfected with either control vector pc or pcLANA construct for additional 48 h. Protein expression were measured by immunoblots. The concentrations of VEGF in culture supernatants were determined using ELISA and cell invasion was measured by the transwell assays. Error bars represent the S.E.M. for 3 independent experiments. ** = *p* < 0.01.

We next aimed to determine which viral proteins are potentially responsible for upregulation of HO-1 in endothelial cells. We previously reported that KSHV-encoded latency-associated nuclear antigen (LANA) alone was sufficient to induce CD147 expression in HUVEC [17, 28]. The LANA protein sequence can be divided into three functional domains: a conserved proline- and serine-rich N-terminal region (domain A), a central region composed of several acidic repeats (domain B), and a conserved C-terminal domain containing a proline-rich region and a region rich in charged and hydrophobic amino acids (domain C) [29]. Both N- and C- terminal domains contain a nuclear localization sequence (NLS, Figure 5A). By using a variety of LANA deletion fragment and full-length constructs, we found that LANA domain A (LANA-A) was sufficient for the upregulation of CD147 and HO-1 expression as full-length LANA did in HUVEC (Figure 5B). We also found that silencing of CD147 by RNAi significantly reduced the expression of HO-1 in LANA-transfected HUVEC (Figure S5). Furthermore, ectopic expression of LANA in HUVEC increased VEGF production and cell invasion, whereas which can be significantly blocked by silencing of HO-1 with specific siRNA (Figure 5C-5E).

### SnPP treatment effectively suppresses TIVE-LTC tumorigenesis *in vivo*

**Figure 6:**
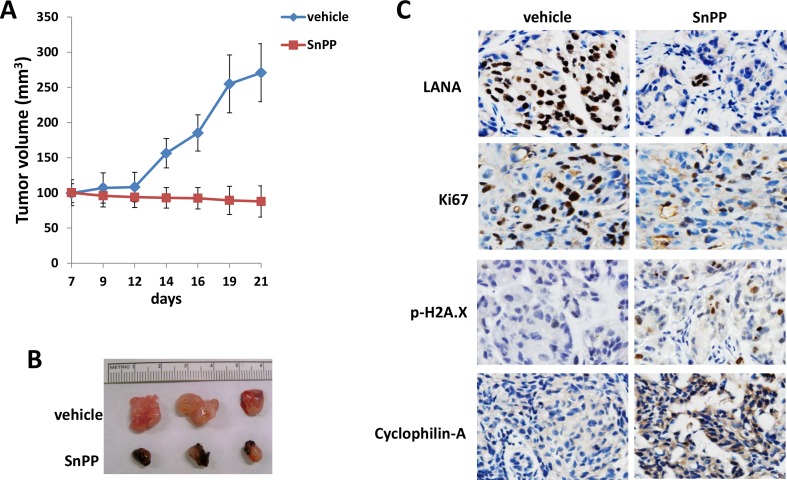
Targeting HO-1 by SnPP effectively suppresses TIVE-LTC tumorigenesis *in vivo* **A.**-**B.** TIVE-LTC (5 × 10^5^ cells 1:1 with growth factor-depleted Matrigel) were injected subcutaneously into the right and left flanks of nude mice (3 mice per group), respectively. When tumors reach 10-15 mm in diameter (∼1.5weeks), mice were received *in situ* subcutaneous injection with either vehicle or SnPP (10 μmol/kg of body weight), 5 days/week. The mice were observed and measured every 2∼3 d for the size of palpable tumors for additional 2 weeks. At the end of experiment, the tumors were excised from the site of injection for subsequent analysis. In SnPP treated mice, some dark substance (SnPP residues) around the tumors were observed when they were excised, which can be easily stripped from tumor tissues. Error bars represent the S.E.M. for one of 2 independent experiments. **C.** Protein expression in tumor tissues from representative mice was measured by immunohistochemistry as described in the Methods.

By using an established KS-like nude mouse model with TIVE-LTC [18, 19], we tested the effects of SnPP on TIVE-LTC tumorigenesis *in vivo*. We injected TIVE-LTC (5 × 10^5^ cells 1:1 with growth factor-depleted Matrigel) subcutaneously into the right and left flanks of nude mice (3 mice per group), respectively. When tumors reached 10-15 mm in diameter (∼1.5weeks), mice received *in situ* subcutaneous injection with either vehicle or SnPP (10 μmol/kg of body weight), 5 days/week. The mice were observed every 2∼3 d and palpable tumors were measured for additional 2 weeks. Our results indicated that SnPP treatment significantly repressed tumor growth in mice while vehicle had no effect (Figure 6A). SnPP treated mice formed significantly smaller tumors when compared to vehicle treated group after 2-week treatment (Figure 6B). Immunohistochemistry analysis results indicated the increased expression of phosphor-H2A.X and Cyclophilin-A, while the reduced expression of LANA and cellular proliferation indicator Ki67 in tumor tissues isolated from representative SnPP-treated mice when compared to those from vehicle-treated mice (Figure 6C).

## DISCUSSION

In the current study, we identify for the first time the upregulation of HO-1 expression by either host CD-147 or viral LANA proteins within endothelial cells. Ongoing work focuses on identifying the mechanisms for upregulation of HO-1 by CD147 either directly (CD147 binding to HO-1 promoter region), or indirectly through other cellular transcriptional factors (e.g. CREB and Nrf2) and/or signaling pathway (e.g. MEK/ERK or PI3K/AKT) [30]. We do not exclude other regulatory mechanisms which may exist, and actually, one very recent study has shown that one of KSHV microRNAs, miR-K12-11, an ortholog of human oncomir miR-155 [31, 32] can induce HO-1 expression from lymphatic endothelial cells (LEC) through directly targeting BACH1 [33], a cellular HO-1 transcriptional repressor [34]. Interestingly, these authors indicate that there are other viral microRNA-independent mechanisms for induction of HO-1 expression in LEC [33]. These data and our findings in the current study suggest a model in which KSHV infection induces HO-1 expression and its downstream activities through multiple viral and host factors, although it still requires further investigation.

As mentioned above, HO-1 can cleave the porphyrin ring-releasing equimolar quantities of CO, free iron, and biliverdin. Free iron then stimulates the production of the iron-scavenging protein, Ferritin, while biliverdin is rapidly reduced to bilirubin by biliverdin reductase [10]. In fact, these HO-1 metabolites have important roles in endothelial cell physiology. For example, CO produced by HO-1 activity has been shown to protect endothelial cells from both CD95/Fas- and tumor necrosis factor (TNF)-mediated apoptosis [35, 36]. In addition, bilirubin together with upregulated Ferritin has been shown to protect endothelial cells from oxidative damage resultant from myriad noxious stimuli [37, 38]. Since the typical “spindle” KS tumor cells are endothelial derived [39], it will be interested to understand how these HO-1 metabolites contribute to the pathogenesis of KSHV-infected endothelial cells and KS development.

One of our findings is that targeting HO-1 by SnPP induces cell death in KSHV-infected endothelial cells through necrosis but not apoptosis, which is different from some previous studies. For example, Marinissen *et al* have reported that SnPP treatment induces endothelial cell apoptosis [14]. However, in this study the authors used vGPCR- or HO-1-transfected Simian virus 40, large T-antigen-immortalized, murine endothelial cells (SVECs) [14], instead of human endothelial cells such as TIVE-LTC we used in the present study. We assume that different cell-lines with genetic modification may cause varied responses to SnPP treatment, although this still requires experimental validation. Therefore, we will treat more primary or immortalized endothelial cell-lines with SnPP to observe its effect.

By using a KS-like nude mouse model, we found that SnPP treatment can effectively suppress TIVE-LTC tumorigenesis *in vivo*. Interestingly, it also greatly reduced LANA expression in the tumor tissues, although the underlying mechanisms remain unknown. However, considering that high dose of SnPP displays some cytotoxicity especially to non-infected endothelial cells such as TIVE (Figure 2A-2B) and HUVEC (data not shown), it might be better to combine SnPP with other chemotherapeutical regimens for clinical treatment or to develop other HO-1 selective inhibitors such as the imidazole-dioxolane compounds [40]. Unlike the metalloporphyrins such as SnPP, these imidazole-dioxolane compounds are selective for the inhibition of HO with minimal effects on other heme-dependent enzymes such as nitric oxide synthase and soluble guanylyl cyclase [40]. Interestingly, some chemotherapeutical agents such as paclitaxel and rapamycin (both of which have been used for KS treatment) [41, 42] have been found to induce HO-1 expression and activities [43, 44]. Therefore, future study will test whether combination of HO-1 inhibitors can reduce tumor burden as well as KS tumor cell resistance to chemotherapy.

## MATERIALS AND METHODS

### Cell culture, reagents and infection protocol

Body cavity-based lymphoma cells (BCBL-1, KSHV^+^/EBV^neg^) were kindly provided by Dr. Dean Kedes (University of Virginia) and maintained in RPMI 1640 medium (Gibco) with supplements as described previously [45]. Telomerase-immortalized human umbilical vein endothelial (TIVE) and KSHV long-term-infected TIVE cells (TIVE-LTC) were cultured as previously described [19]. Human umbilical vein endothelial cells (HUVEC) were grown in DMEM/F-12 50/50 medium (Cellgro) supplemented with 5% FBS. All cells were incubated at 37°C in 5% CO_2_. All experiments were carried out using cells harvested at low (< 20) passages. SnPP and the pan-caspase inhibitor Z-VAD-FMK were purchased from Sigma. To obtain KSHV for infection experiments, BCBL-1 cells were incubated with 0.6 mM valproic acid for 6 days, and purified virus was concentrated from culture supernatants; infectious titers were determined as described previously [46]. For overexpression of CD147, HUVEC were transduced as previously described with a recombinant adenoviral vector (MOI ∼ 10) encoding CD147 (AdV-CD147), or a control vector (AdV), for 48 h prior to subsequent analysis [47].

### RNA interference and plasmid transfection

*CD147* or *HO-1* ON-TARGET plus SMART pool siRNA, or negative control siRNA (n-siRNA) (Dharmacon), were delivered using the DharmaFECT transfection reagent according to the manufacturer's instructions. For plasmid transfection, HUVEC were transfected with control vector pcDNA3.1, pcDNA3.1-LANA (pcLANA) or LANA deletion fragments (pcLANA-A, pcLANA-AB, pcLANA-AC, pcLANA-BC and pcLANA-C) [28] in 12-well plates for 48 h using Lipofectamine 2000 (Invitrogen) according to the manufacturer's instruction. Transfection efficiency was determined through co-transfection of a lacZ reporter construct and quantified as described previously [46].

### Immunoblotting

Total cell lysates (20μg) were resolved by 10% SDS-PAGE, transferred to nitrocellulose membranes, and immunoblotted with antibodies for CD147 (BD), HO-1, p-H2A.X/t-H2A.X, Cyclophilin-A, HMGB1 (Cell Signaling) and β-Actin (Sigma) for loading controls. Immunoreactive bands were identified using an enhanced chemiluminescence reaction (Perkin-Elmer), and visualized by autoradiography.

### Immunofluorescence

Cells were incubated in 1:1 methanol-acetone at −20°C for fixation and permeabilization, then with a blocking reagent (10% normal goat serum, 3% bovine serum albumin, and 1% glycine) for an additional 30 min. Cells were then incubated for 1 h at 25°C with 1:400 dilution of a rabbit anti-p-H2A.X, anti- Cyclophilin-A or anti-HMGB1 antibody (Cell Signaling) followed by 1:200 dilution of a goat anti-rabbit secondary antibody conjugated to Texas Red or Alexa 488 (Invitrogen). For identification of nuclei, cells were subsequently counterstained with 0.5 mg/mL 4′,6-diamidino-2-phenylindole (DAPI; Sigma) in 180 mM Tris-HCl (pH 7.5). Slides were washed once in 180 mM Tris-HCl for 15 minutes and prepared for visualization using a Leica TCPS SP5 AOBS confocal microscope.

### HO enzymatic activity

Crude endothelial cell protein extracts were prepared as previously described [13, 20]. Briefly, following 24-h incubation in complete media alone or with SnPP, monolayers were rinsed with PBS and scraped directly into 300 μL sonication buffer (0.25 M sucrose, 20 mM Tris-HCl, 50 μg/mL Pefabloc SC, 4 μg/mL leupeptin; pH 7.4) sonicated on ice 2 times for 30 s and centrifuged for 20 min at 18,000 g. The protein concentration of the resultant supernatant was determined using BCA as described above. HO activity was measured by the spectrophotometric determination of bilirubin production as described elsewhere [13, 20]. HO activity was reported as picomoles of bilirubin produced per milligram endothelial cell protein extract per hour.

### Cometassay

The DNA damage was evaluated by using the Reagent Kit for Single Cell Gel Electrophoresis Assay/CometAssay (Trevigen), according to the manufacturer's instructions. The slides were viewed by using epifluorescence microscopy. The tail moment was calculated from 100 cells collected per single measurement by utilizing specialized comet software included in the Automated Comet Assay System (Loats Associates Inc).

### qRT-PCR

Total RNA was isolated using the RNeasy Mini kit (QIAGEN), and cDNA was synthesized from equivalent total RNA using a SuperScript III First-Strand Synthesis SuperMix Kit (Invitrogen) according to the manufacturer's instructions. Primers used for amplification of target genes are displayed in Supplemental Table 1. Amplification was carried out using an iCycler IQ Real-Time PCR Detection System, and cycle threshold (Ct) values were tabulated in duplicate for each gene of interest in each experiment. “No template” (water) controls were used to ensure minimal background contamination. Using mean Ct values tabulated for each gene, and paired Ct values for *β-actin* as a loading control, fold changes for experimental groups relative to assigned controls were calculated using automated iQ5 2.0 software (Bio-Rad).

### Cell proliferation and apoptosis assays

Cell proliferation was measured by using the WST-1 assays (Roche) according to the manufacturers' instructions. For apoptosis assays, the FITC-Annexin V and propidium iodide (PI) Apoptosis Detection Kit I (BD Pharmingen) was used. Samples were analyzed on a FACS Calibur 4-color flow cytometer (BD Bioscience).

### ELISA

Concentrations of VEGF in culture supernatants were determined using human VEGF-A (Pierce Biotechnology) ELISA kits according to the manufacturers' instructions.

### Transwell invasion assays

Matrigel Invasion Chambers (BD) were hydrated for 4 h at 37°C with culture media. Following hydration, media in the bottom of the well was replaced with fresh media, then 2×10^4^ HUVEC or TIVE-LTC were plated in the top of the chamber. After 24 h, cells were fixed with 4% formaldehyde for 15 min at room temperature and chambers rinsed in PBS prior to staining with 0.2% crystal violet for 10 min. After washing the chambers, cells at the top of the membrane were removed and cells at the bottom of the membrane counted using a phase contrast microscope. Relative invasion was determined for cells in experimental groups as follows: relative invasion = # invading cells in experimental group / # invading cells in control groups.

### Soft agar assays

A base layer containing 0.5% agarose medium and 5% FCS was poured into six-well plates. Then, 10,000 cells were mixed with 0.4% agarose in Earl's minimal essential medium (EMEM) containing 5% FCS to form a single-cell suspension. After being seeded, the plates were incubated for 2 weeks. Colonies were stained with 0.005% crystal violet and photographed under a phase-contrast microscope (Leica DFC320).

### KS-like nude mouse model

Cells were counted and washed once in ice-cold PBS, and 5 × 10^5^ TIVE-LTC in 50 μL PBS plus 50 μL growth factor-depleted Matrigel (BD Biosciences) were injected subcutaneously into the two flanks of nude mice (Jackson Laboratory). The mice were observed and measured every 2∼3 d for the presence of palpable tumors. When tumors reach 10-15 mm in diameter (∼1.5weeks), mice were received *in situ* subcutaneous injection with either vehicle or SnPP (10 μmol/kg of body weight dissolved in 0.1 N NaOH in PBS, pH 7.5), 5 days/week. At the end of experiment, the tumors were excised from the site of injection for subsequent analysis such as immunohistochemistry. All protocols were approved by the LSUHSC Animal Care and Use Committee in accordance with national guidelines.

### KS tumor tissues from HIV+ patients and immunohistochemistry

KS tissues from HIV-infected patients were provided by the LSUHSC HIV Outpatient (HOP) Clinic and Biospecimens Bank (LSUHSC IRB approved No. 8079). Formalin-fixed, paraffin-embedded tissues were microtome-sectioned to a thickness of 4 uM, placed on electromagnetically charged slides (Fisher Scientific), and stained with hematoxylin & eosin (H&E) for routine histologic analysis. Immunohistochemistry was performed using the Avidin-Biotin-Peroxidase complex system, according to the manufacturer's instructions (Vectastain Elite ABC Peroxidase Kit; Vector Laboratories). In our modified protocol, sections were deparaffinized in xylene and re-hydrated through a descending alcohol gradient. For non-enzymatic antigen retrieval, slides were heated in 0.01 M sodium citrate buffer (pH 6.0) to 95°C under vacuum for 40 min and allowed to cool for 30 min at room temperature, then rinsed with PBS and incubated in MeOH/3% H_2_O_2_ for 20 min to quench endogenous peroxidase. Slides were then washed with PBS and blocked with 5% normal goat serum in 0.1% PBS/BSA for 2 h at room temperature, then incubated overnight with indicated antibody at 1:200-1:400 dilution in 0.1% PBS/BSA. The following day, slides were incubated with appropriate secondary antibody at room temperature for 1 h, followed by avidin-biotin peroxidase complexes for 1 h at room temperature. Finally, slides were developed using a diaminobenzidine substrate, counterstained with hematoxylin, dehydrated through an ascending alcohol gradient, cleared in xylene, and coverslipped with Permount. Images were collected using an Olympus BX61 microscope equipped with a high resolution DP72 camera and CellSense image capture software.

### Statistical analysis

Significance for differences between experimental and control groups was determined using the two-tailed Student's *t*-test (Excel 8.0), and *p* values < 0.05 and/or < 0.01 were considered significant.

## SUPPLEMENTARY MATERIAL FIGURES AND TABLE


