# Supplementary material for: Role of heme oxygenase-1 in the pathogenesis and tumorigenicity of Kaposi's sarcoma-associated herpesvirus
Source: Oncotarget. 2016 Feb 7;7(9):10459–71. doi: 10.18632/oncotarget.7227 (PMC4891132; doi:10.18632/oncotarget.7227)
Supplement: Supplementary file 1 [file oncotarget-07-10459-s001.pdf]

# Role of heme oxygenase-1 in the pathogenesis and tumorigenicity of Kaposi's sarcoma-associated herpesvirus

**Supplementary Material**

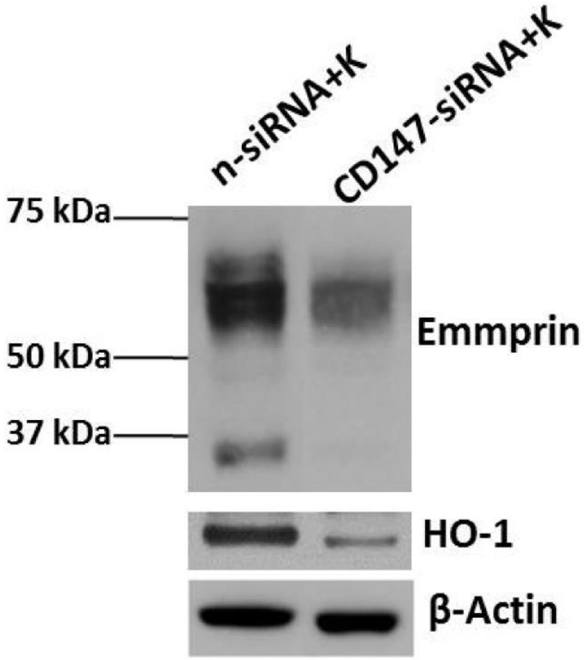


**Figure S1: Targeting CD147 by RNAi reduces HO-1 expression in KSHV-infected HUVEC.** HUVEC were transfected with negative control siRNA (n-siRNA) or *CD147*-siRNA for 48 h, then infected by purified KSHV (MOI ~ 10) for additional 48 h. Protein expression was measured by immunoblots.

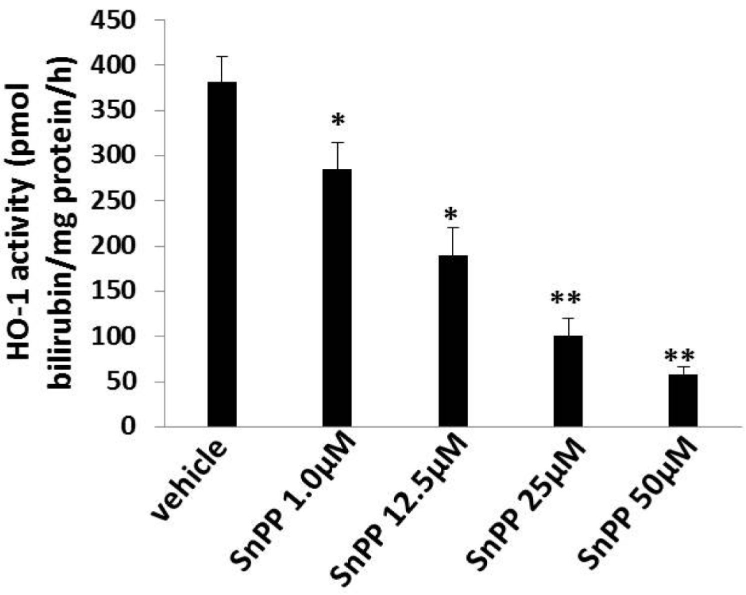


**Figure S2: Measurement of HO activity in TIVE-LTC cell extracts.** TIVE-LTC were incubated with vehicle or indicated concentrations of SnPP for 24 h. HO activity in TIVE-LTC cell extracts was determined by spectrophotometric measurement of bilirubin production as described in the Methods. Error bars represent the S.E.M. for 3 independent experiments. * = p<0.05; ** = p<0.01.

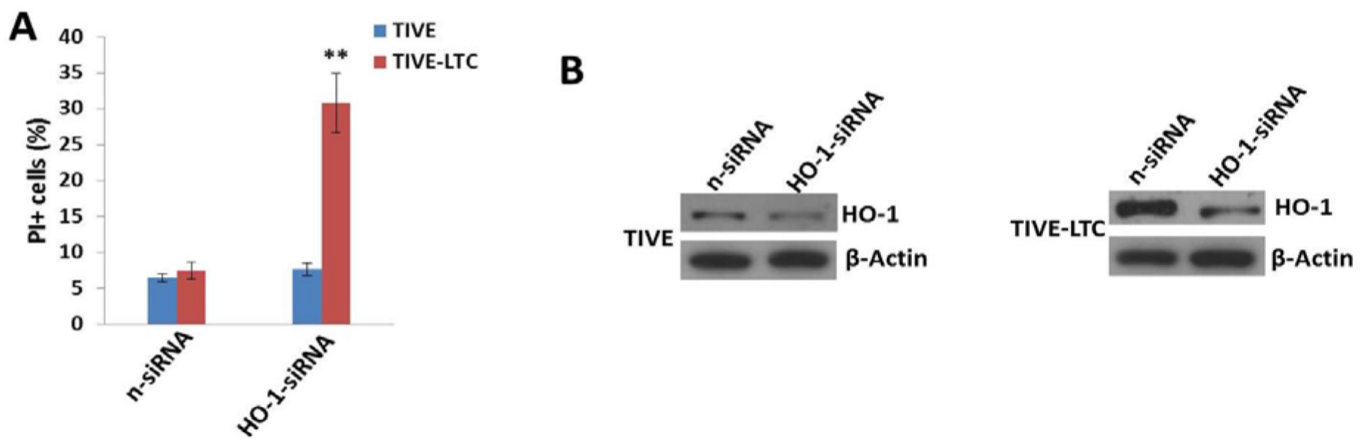


**Figure S3: Targeting HO-1 by RNAi induces TIVE-LTC cell death.** (**A**) TIVE-LTC and parental TIVE cells were transfected with negative control siRNA (n-siRNA) or *HO-1*-siRNA for 48 h, then cell viability was measured by Annexin V-PI staining and flow cytometry analysis. (**B**) Protein expression was measured by immunoblots. Error bars represent the S.E.M. for 3 independent experiments. ** = p<0.01.

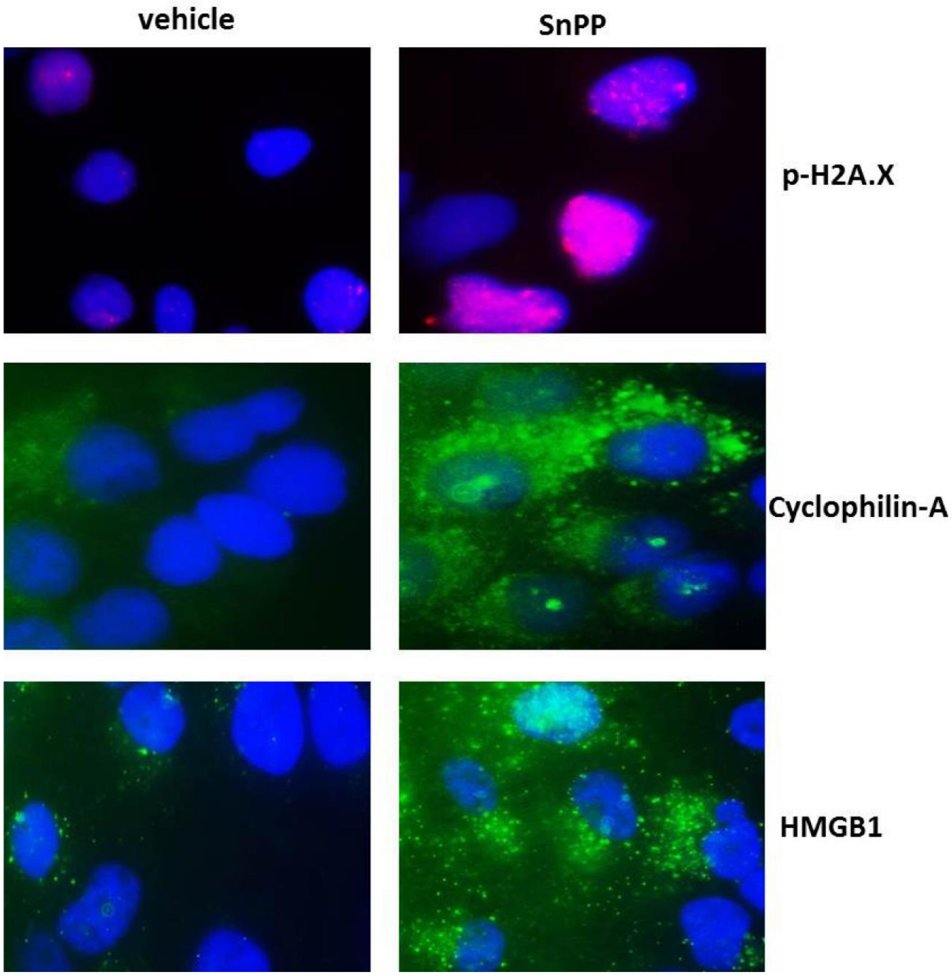


**Figure S4: SnPP treatment increases the expression of DNA damage and necrosis markers within TIVE-LTC.** TIVE-LTC were incubated with vehicle or 50 µM of SnPP for 24 h, then protein expression was measured by immunofluorescence and representative images were shown.

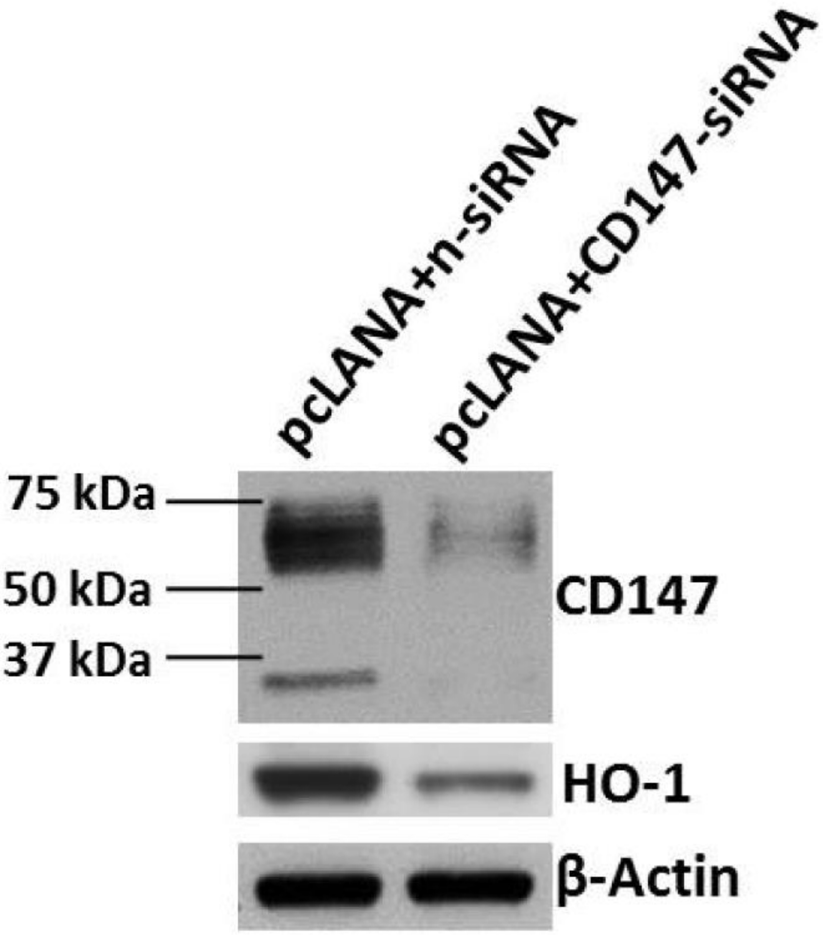


**Figure S5: Targeting CD147 by RNAi reduces HO-1 expression in LANA-transfected HUVEC.** HUVEC were transfected with negative control siRNA (n-siRNA) or *CD147*-siRNA for 48 h, then transfected with LANA construct (pcLANA) for additional 48 h. Protein expression was measured by immunoblots.

**Supplemental Table 1. Primer sequences for qRT-PCR in this study.**

| **Gene** | **Sequences (5' ⟶ 3')** | |
|---|---|---|
| *HO-1* | *sense* | *TTTGAGGAGTTGCAGGAGC* |
| | *antisense* | *AGGACCCATCGGAGAAGC* |
| *VEGFR1* | *sense* | *GACTAGATAGCGTCACCAG* |
| | *antisense* | *AATACTCCGTAAGACCACA* |
| *VEGFR2* | *sense* | *AAAGGGTGGAGGTGACTG* |
| | *antisense* | *GACATAAATGACCGAGGC* |
| *β-actin* | *sense* | *GGAAATCGTGCGTGACATT* |
| | *antisense* | *GACTCGTCATACTCCTGCTTG* |
